# An Intrusion Detection System for the Protection of Railway Assets Using Fiber Bragg Grating Sensors

**DOI:** 10.3390/s141018268

**Published:** 2014-09-29

**Authors:** Angelo Catalano, Francesco Antonio Bruno, Marco Pisco, Antonello Cutolo, Andrea Cusano

**Affiliations:** Optoelectronic Division-Department of Engineering, University of Sannio, Corso Garibaldi 107, Benevento 82100, Italy; E-Mails: acatalano@unisannio.it (A.C.); frbruno@unisannio.it (F.A.B.); pisco@unisannio.it (M.P.); cutolo@unisannio.it (A.C.)

**Keywords:** intrusion detection, optical fiber, sensors, Fiber Bragg Grating, security, railway

## Abstract

We demonstrate the ability of Fiber Bragg Gratings (FBGs) sensors to protect large areas from unauthorized activities in railway scenarios such as stations or tunnels. We report on the technological strategy adopted to protect a specific depot, representative of a common scenario for security applications in the railway environment. One of the concerns in the protection of a railway area centers on the presence of rail-tracks, which cannot be obstructed with physical barriers. We propose an integrated optical fiber system composed of FBG strain sensors that can detect human intrusion for protection of the perimeter combined with FBG accelerometer sensors for protection of rail-track access. Several trials were carried out in indoor and outdoor environments. The results demonstrate that FBG strain sensors bonded under a ribbed rubber mat enable the detection of intruder break-in via the pressure induced on the mat, whereas the FBG accelerometers installed under the rails enable the detection of intruders walking close to the railroad tracks via the acoustic surface waves generated by footsteps. Based on a single enabling technology, this integrated system represents a valuable intrusion detection system for railway security and could be integrated with other sensing functionalities in the railway field using fiber optic technology.

## 1. Introduction

To meet the ever-increasing demand for improved security, worldwide research on intrusion detection sensing systems has grown significantly. In particular, intrusion-sensing systems are becoming much more attractive to the transport sector in which increased protection for passengers, assets, and infrastructure is greatly needed. The usage and implementation of intrusion detection systems in railway scenarios would allow monitoring and protection of railway tunnels, level crossings, train depots, and other similar areas featuring railway scenarios. In these cases, intrusion detection systems could be used to protect properties from theft or vandalization, leading to significant cost savings.

Conventional technologies for intrusion detection systems that are installed to complement human guards (and video cameras systems) typically involve microwave sensors, electric field sensors, ported coaxial cables, and infrared sensors [1]. Among these, the electronics-based technologies, even if well assessed, are inefficient and unsuitable for railway scenarios because they can be adversely affected by the electromagnetic interferences that are inevitably associated with the train transit. An additional difficulty lies in the increased complexity required to multiplex a large number of sensors, which affects the efficiency of their use for large-area protection. Infrared (or photoelectric) sensors instead are sensitive to dust and weather conditions, and their operation is also limited to straight-line detection.

In contrast to these conventional technologies, optical fiber sensors can be considered as a valid option due to the intrinsic advantages associated with their use. Optical fibers sensors offer immunity to electromagnetic interference as well as high sensitivity, compactness, remote sensing ability, and stability in harsh environments. Furthermore, the optical fiber plays the dual role of sensor and communication medium, which strongly reduces the complexity of the telemetry systems.

Among the fiber optic sensors, Fiber Bragg Grating (FBGs) sensors also offer high multiplexing capability, versatility, and simplicity of use, allowing the deployment of a cost-effective interrogation strategy. Additionally, FBGs have been employed previously for perimeter security systems. In particular, Rao *et al.* demonstrated an optical fiber fence based on FBGs [2]. Successively, Wu *et al.* reported an FBG-based fiber-optic fence and developed a method based on the autocorrelation characteristics of the signals to improve the system performances by reducing the number of nuisance alarms [3]. Nevertheless, even if sensorized fences have demonstrated their effectiveness in protecting a perimeter [4], the use of sensing fences in a railway scenario is not an attractive strategy. In fact, fences represent a physical barrier that inhibits personnel passage (even when it should be allowed) and obstructs the rail tracks and thus the transit of trains.

**Figure 1.**
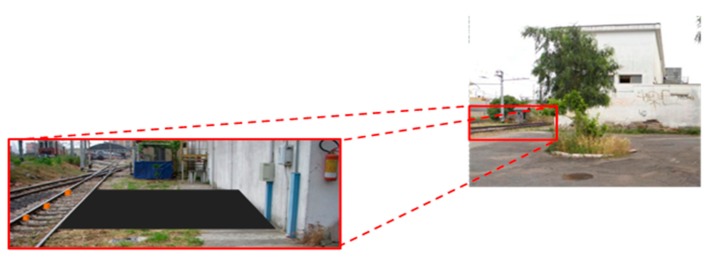
Ponticelli service area (Ente Autonomo Volturno-ex Circumvesuviana-Railway-Naples, Italy).

Alternatively, Allwood *et al.* [5,6] investigated the possibility of using FBGs as acoustic emission receivers for in-ground intrusion detection systems. Laboratory trials showed that an FBG sensor embedded within a thick concrete slab can be used effectively as an in-ground acoustic sensor to detect the acoustic emissions associated with a human walking on a concrete surface. Similarly, Zhang *et al.* demonstrated a FBG seismic sensor in a cantilever configuration for military vehicle detection [7]. These reports follow previous and promising experimental studies carried out with conventional technologies in which the intruder is detected by measuring the seismic vibrations or acoustic signals produced by contact between the foot and the ground/floor [8–10]. In this paper, we report on the development of an intrusion detection system based on FBGs that is well suited for protecting railway areas from unauthorized access. Specifically, this method combines the advantages of solutions involving the sensorization of a perimeter with the advantages of solutions that exploit the propagation of the vibrations induced by intruder activities. Therefore, to protect railway areas, we propose the use of a hybrid solution composed of a sensorized mat for ground perimeter protection and an accelerometer sensing system for protection of rail tracks. Most of the railways scenarios that must be protected share a common feature, namely, the presence of access zones (for passengers, operators, *etc*.) interleaved by rail tracks (for train transit) that cannot be practically obstructed with physical barriers. As an example, we refer to a specific case, *i.e*., the Service Area of Ente Autonomo Volturno Railway in Naples (Italy). As shown in Figure 1, the entry of the service area displays regular ground on which a sensing mat could be installed and also the presence of rail tracks, which should be covered by the accelerometer sensing system.

In the following section, we briefly describe the principle of operation of the proposed sensing system. In the successive sections, we describe the methodology and the experimental results for indoor lab experimental activities carried out for the development of an FBG sensorized mat and outdoor experimental activities used for the development of a fiber-optic accelerometer sensing system.

## 2. Principle of Operation

### 2.1. Sensorized Mat

**Figure 2.**
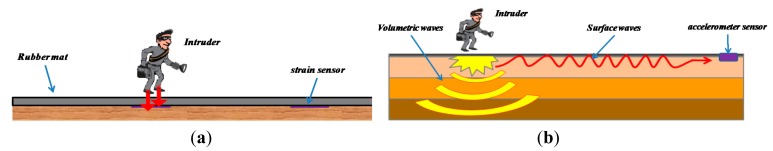
(**a**) Operational principle of the sensorized mat and (**b**) of the accelerometer sensing system.

The intrusion detection system for walkable access to a protected site is composed of a mat sensorized with FBG strain sensors, as schematized in Figure 2a. An intruder walking on the mat represents a load on the upper surface of the mat. The applied pressure leads to a deformation of the mat, according to its elastic properties, and an FBG bonded on the lower surface of the mat senses the strain associated with the load by converting the strain into a Bragg wavelength shift. By deploying a net of properly spaced FBGs under the mat, detection of a human walking on the mat can be achieved by monitoring the respective Bragg wavelengths.

### 2.2. Accelerometer Sensing System under the Rail Track

The fiber-optic sensing system for rail track access protection is composed of two accelerometers based on FBG technology, one for each rail track. An intruder moving on the ground represents a vibration source. The seismic/acoustic surface waves propagate omni-directionally in the ground. Because the surface wave couples to the rail tracks, FBG accelerometers installed under the rail tracks are able to detect the acoustic waves generated by the footsteps of a human. Thus, a person passing through a perimeter that must be protected via the rail tracks can be detected by monitoring the Bragg wavelength shifts of the FBG-based accelerometers (see Figure 2b).

## 3. Methodology

In this section, we report on the methodology of the experimental tests, which are divided according to strain and acceleration measurement trials.

### 3.1. Strain Measurement Trials

Preliminary lab activities were performed to characterize the elastic response of the sensorized mat in the presence of applied static loads and to determine the performance in terms of the sensitivity curve, response times, and required number of sensors per area unit.

To maximize the strain induced by the applied load, we selected rubber as the mat material due to its low Young modulus (10–100 MPa [11]). The mat thickness was as thin as 3 mm, and it was also patterned with ridges with a 1-mm depth on the lower side.

The “ribbed” mat allows for optical fiber placement and easy FBG sensor installation. Additionally, the possibility of damage or breakage of the optical fiber is minimized because the optical fiber is not exposed directly to the floor surface but is protected by the ribs of the mat.

Once the material and geometry of the mat were identified, a 1570-nm FBG was glued onto the lower surface of the ribbed rubber mat using cyanoacrylate. The FBG wavelength shifts are detected using a commercial FBG interrogator (Micron Optics Interrogator-SM125).

To characterize the elastic response of the sensorized mat, a sequence of cast iron discs (10 kg-10 kg-20 kg-20 kg) was applied on the mat one-by-one, with time intervals of five minutes corresponding to the FBG sensor. With the same time steps, all of the discs were singularly removed to recover the initial condition. During the tests, to assure uniformity of the applied pressure on the mat surface, an iron plate with a circular base, a diameter of 10 cm, and a thickness of 1 cm was positioned between the mat and the plates corresponding to the measured point. The size of the iron plate was selected such that its area was comparable with the area covered by a sole of a foot.

A similar analysis was performed by applying a static load at different distances from the FBG to retrieve the influence zone of each sensor, *i.e*., the sensing area for a single FBG.

Based on the results of the performed characterizations, we deployed the FBG sensors in a network and verified the ability of the sensing system to detect an intruder walking on the mat.

Finally, we investigated the possibility of improving the performances of the sensorized mat by increasing the size of the sensing area for a single FBG (detection zone) through the use of a “rigid” plate superimposed on the rubber mat.

### 3.2. Acceleration Measurement Trials

Several trials were performed at the Maddaloni-Marcianise freight station in Italy to investigate the ability of the proposed sensing system to detect the presence of an intruder walking close to the rail tracks. The two accelerometers used in the fiber-optic sensing system for rail track access protection are both one-axis commercial accelerometers (os7100, Micron Optics, Atlanta, GA, USA) based on FBG technology. These accelerometers feature a flat responsivity (from DC to 300 Hz) of ∼16 pm/g and a resonance frequency of ∼700 Hz.

**Figure 3.**
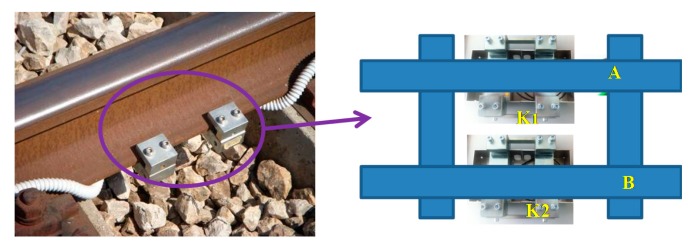
Packages containing the FBG accelerometer positioned under the rail track. Inset: Schematization of the rail track with the two FBG accelerometers fixed under the rails and labeled K1 and K2.

The accelerometers were positioned under the tracks according to the perimeter to be protected using proper packaging with the axis of measurement perpendicular to the ground plane, as shown in Figure 3. The packages were designed for installation without damage to the rail track (*i.e*., without drilling and welding to the rail tracks) and obviously without removal of the rail tracks [12]. For interrogation of the system, we used a Smart Scan Fibre Interrogator (Smart Fibres Limited, Bracknell, UK) set to acquire the wavelength samples with a frequency of 2.5 kHz placed in a control room located at a far distance (400 m) from the measurement point.

During the outdoor trials, an 80-kg male (the intruder) walks near the tracks, covering a total distance of 20 m. In particular, he begins by walking parallel to the rail tracks from a distance of 10 m from the sensors, subsequently walks up to the sensors, and walks away for an additional 10 m. We repeated these tests by changing the relative position between the intruder and the rails to investigate the influence zone of the accelerometer sensors.

## 4. Experimental Results and Discussions

The following paragraphs report a selection of results that are representative of the performed tests and are divided according to strain and acceleration measurements.

### 4.1. Strain Measurements

**Figure 4.**
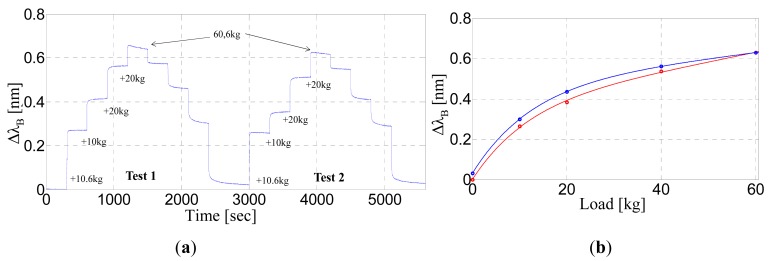
(**a**) Bragg wavelength shift *versus* time under the application of different weight plates; (**b**) Sensitivity curve: Ascent (Red) and the descent (Blue).

The FBG response *versus* time is shown for different applied weights in Figure 4a. The figure clearly shows that the Bragg wavelength shift (Δλ_B_) increases according to the positioning of each weight as it returns back to the initial position after the successive weight removal. The response times are as low as one second. In particular, for the FBG response shown in Figure 4a, the maximum value of the response time (which is evaluated as the time from 10% to 90% of the steady state values) is 0.6 s.

The observed wavelength shifts are repeatable with an error of up to 15% with respect to the steady-state wavelength value. Nevertheless, the Bragg wavelength does not completely recover to the initial value after 5 min from the removal of all weights. As evident in Figure 4b, indeed, the sensitivity curve presents a hysteretic behavior that can be attributed to the physical and mechanical properties of the natural rubber itself. The calculated value for the elastic hysteresis (the integral area between the upper and the lower curve) is approximately 1857 kg·pm, whereas the linear sensitivity, which is calculated in the limit of the zero-load condition, is approximately 33.2 pm/kg. Such sensitivity would allow the detection of a weight positioned on the FBG of as low as 3 kg (assuming a wavelength resolution of 1 pm). It is worth noting that intruder detection does not require such low resolution. However, in a practical situation, the intruder step could occur at a distance from the FBG, and therefore, it is of crucial importance to investigate the FBG response when the load is not applied directly to it.

**Figure 5.**
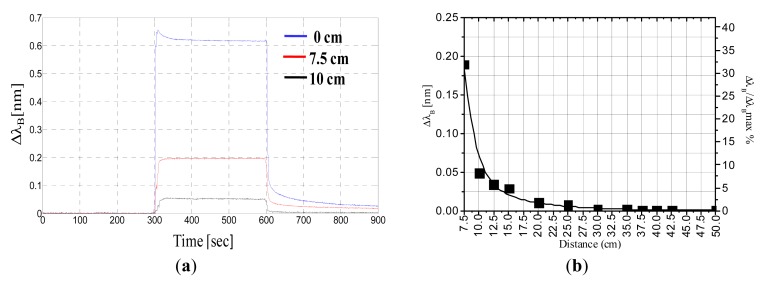
(**a**) Time response of the Bragg wavelength shift at difference distances from the FBG; (**b**) Bragg wavelength shift *versus* distance from the FBG fitted with an exponential decay.

To this end, we applied a 60-kg static load at difference distances from the FBG. Figure 5a shows the time response of the Bragg wavelength shift when the load is applied to the sensor, at distances of 7.5 cm and 10 cm. As shown in Figure 5a, the FBG still responds to applied loads that are not strictly positioned on it, but the amplitude of the wavelength shift obtained with the same weight decreases rapidly with distance from the FBG. Figure 5b shows the Bragg wavelength shift *versus* the distance from the FBG, and in the same plot (on the right *y*-axis), we also report the Bragg wavelength shift normalized to its maximum value. As highlighted by the exponential fitting, the wavelength shift strongly decays for increasing distances and reaches less than 10% of its initial value at a distance of 10 cm. In particular, the shift is as low as 5 pm when the weight is located 25 cm from the FBG. We used this value as a threshold to establish the limit of operation of a single FBG. Consequently, we estimated that the required number of sensors per area unit required to detect a walking human using a sensorized mat is four FBG units per square meter.

**Figure 6.**
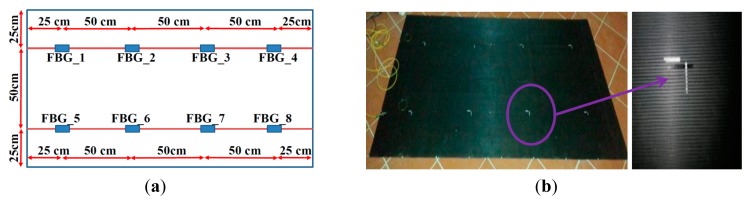
(**a**) Schematic of the sensorized rubber mat; (**b**) Picture of the sensorized mat (Inset: FBGs glued under the ribbed rubber mat).

Based on these results, we fixed eight FBGs equally spaced in a square array configuration on the ribbed surface of a mat with an area of 1 × 2 m^2^ and a thickness of 3 mm, as shown in Figure 6a. Figure 6b displays a picture of the mat. The placement of the FBGs in a regular array is carried out in such a manner that all of the surface area is covered, in terms of detection capability, by at least one FBG. The farthest point from the FBGs (located in the middle of the diagonal of the square with four FBGs at the vertices) is located at a distance of 35 cm at which a wavelength shift of approximately 2 pm is predicted (the interrogation system resolution is 1 pm).

We performed several walking tests on the mat and found that the ribbed rubber mat sensorized with FBGs is always able to detect a human walking on the mat and is thus suitable to reveal “intrusion” through the protected perimeter.

**Figure 7.**
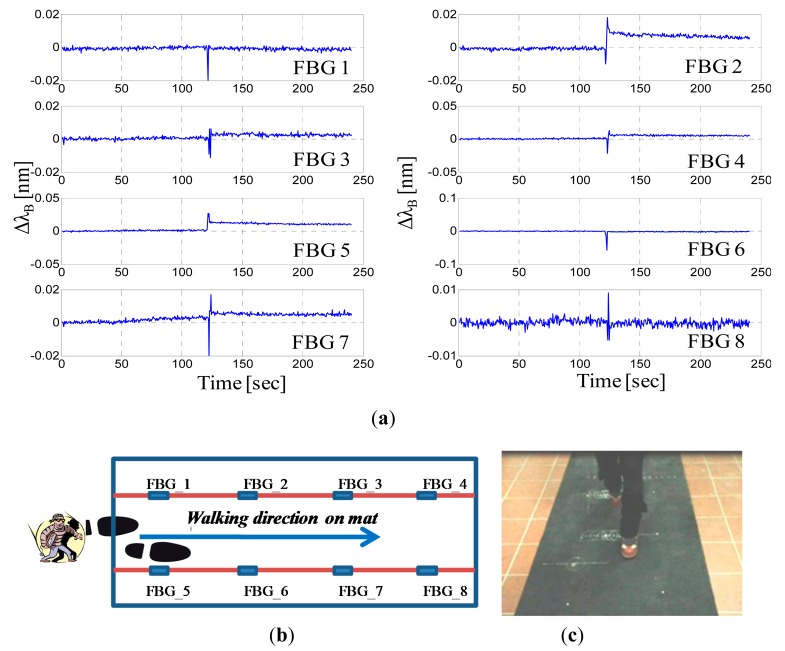
(**a**) Bragg wavelength shift *versus* time; (**b**) Schematic of the intruder's crossing pattern; (**c**) Photo of the walking test on the mat.

Figure 7a presents the wavelength shifts of all eight FBGs recorded during the walk of a 50-kg woman wearing sneakers and crossing all over the mat at the center (see Figure 7b,c). Even if all of the steps fall essentially at the center of the mat, *i.e*., at the maximum distance from the FBGs, each step is easily detected by the nearest FBGs. Indeed, human footsteps can be clearly determined by observing the Bragg wavelength shifts, and each FBG response is characterized by a peak due to an intruder footstep. The maximum amplitudes of the wavelength shifts perceived by each FBG are approximately 20–50 pm, which are much higher (one order of magnitude) than the responses that we observed when we applied a 60-kg static load at a distance of 35 cm from the FBG.

Actually, the load induced by a walking intruder is generally different from the previously applied static loads. Indeed, the FBG response is primarily ruled by the resulting “pressure” applied, and thus, we can expect different responses with the same applied weight if different contact surfaces occur between the foot and the mat. Basically, the static load can be viewed as a worst-case scenario because all of the weight is uniformly distributed on the iron plate in this case. In contrast, when a human walks, the weight is concentrated first on the heel, next on the entire sole, and finally, on the toe of the foot.

**Figure 8.**
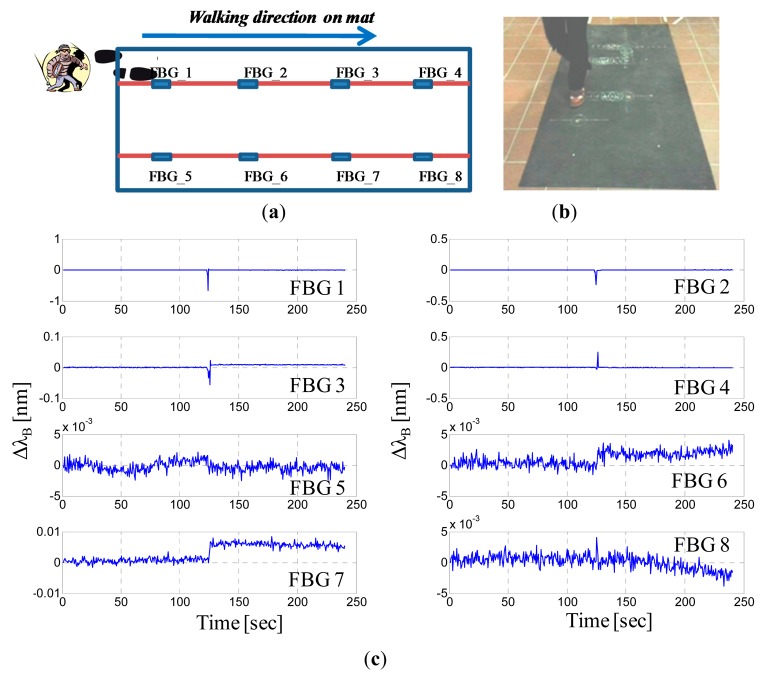
(**a**) Schematic of the intruder's crossing pattern; (**b**) Photo of the walking test on the mat; (**c**) Bragg wavelength shift *versus* time.

As confirmation of this circumstance, Figure 8 shows the wavelength shifts of all eight FBGs recorded during the walk of the same woman (weight of 50 kg and wearing sneakers) crossing all over the mat on the upper side (see Figure 8a,b). The nearest FBGs clearly respond to the footsteps with excursions of up to 500 pm and greater, but it is also interesting to note that the farthest FBGs (on the lower side) slightly respond to the walking woman. Wavelength shifts of approximately 1–5 pm can be observed for the FBGs numbered 6 and 7, even if they are located approximately 50 cm away from the steps. Additionally, it is worth noting that the peaks are not simultaneous, but they are slightly time shifted. Specifically, the maximum absolute wavelength shift for the FBG1 occurs at the 124 s, followed by the maximum shift for the FBG2 at the 124.5 s, for the FBG3 at the 125.5 s and for the FBG4 at the 126 s. By assuming that the wavelength shift achieves its maximum when the foot falls in the detection area of the corresponding sensor, it is possible thus to localize the intruder on the mat and to read out the crossing pattern of a single intruder as well as its speed of crossing. Of course, to this end, the data coming from all FBGs must be properly correlated and analyzed.

**Figure 9.**
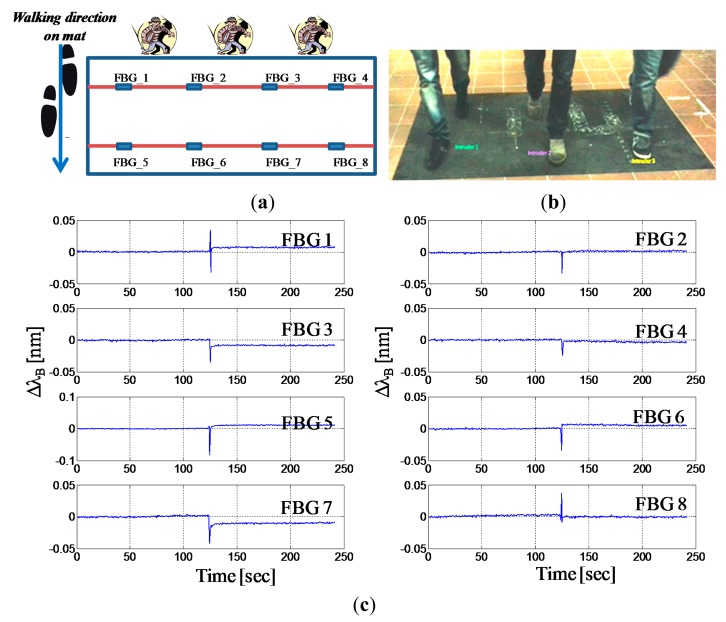
(**a**) Schematic of the intruder's crossing pattern; (**b**) Photo of the walking test on the mat; (**c**) Bragg wavelength shift *versus* time for the eight FBGs.

We performed also experimental tests on the sensorized mat in which multiple intruders are accessing to the protected perimeter. Figure 9a,b show respectively the schematic of the intruders' crossing pattern and a picture of the experimental test featured by three intruders which walk simultaneously over the mat along its short side. Figure 8c presents the wavelength shifts of all eight FBGs during the multiple access. All intruders footsteps are easily detected by the closest FBGs. The maximum amplitude of the wavelength shift of the FBGs ranges approximately from 25 up to 80 pm, mainly in dependence on the proximity of the foot with respect to the sensor (rather than the intruder weight). It can be also noted, by analyzing the peaks' time positions, that the peaks associated to the first raw of FBGs (FBG1-FBG4) are simultaneous (below 1 s) and precede approximately one second the successive peaks associated to the FBGs (FBG5-FBG8).

By taking advantage of the spatial distribution of the sensors or the simultaneity of different FBG responses, in principle, thus it is possible to infer the presence of multiple intruders. Obviously from the collective response of the FBGs is not trivial or always possible to retrieve the intruders number as well as their direction. However, it is worth remarking that the system demonstrated again to be able to reveal the presence of an intrusion.

**Figure 10.**
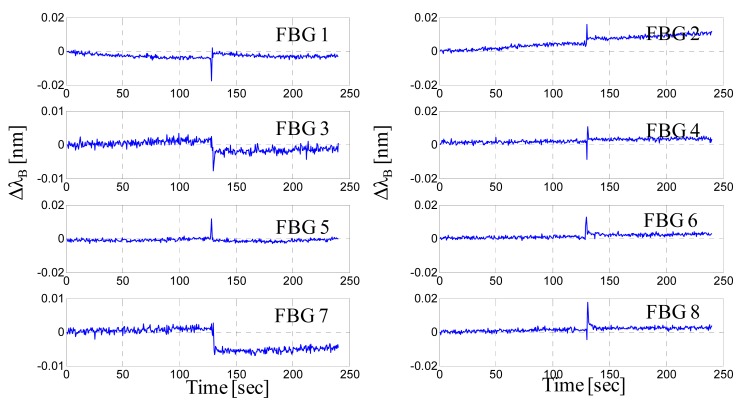
Bragg wavelength shift *versus* time for the eight FBGs.

**Figure 11.**
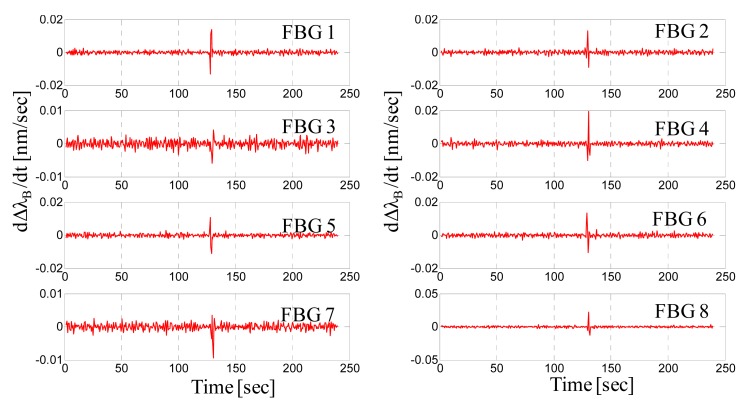
Derivative of the Bragg wavelength shift *versus* time for the eight FBGs.

Once the proper functioning of the sensing system is established for intrusion detection, we focused attention on the alarm generation by privileging some basic aspects specific to the proposed sensing system. Until this point, we recognized the FBG response using the Bragg wavelength shift created by intruder steps, but the Bragg wavelength shift cannot be considered as a valid observable signal. The effects of the elastic hysteresis as well as the daily thermal cycles (drifts) can affect the Bragg wavelength in a “slow” temporal regime. Therefore, to neglect these effects, a signal processing strategy must be adopted based on the comparison of the derivative of the Bragg wavelength with respect to a predetermined threshold. To exemplify this issue, Figure 10 shows an 80-kg man walking and crossing all over the mat at the center. We observe a slight drift in the wavelength response of FBG2 (still recovering from a previous test) as well as the partial recovery of the FBG7 after the step. In contrast, the derivative of the Bragg wavelength still highlights the same steps but neglects the slow terms (Figure 11).

These results essentially indicate that a smart data processing technique must be applied to judge (and assign) an alarm based not only on the wavelength shift but also on the derivative of the Bragg wavelength's shift. This caution is necessary in order to face specific effects, such as the elastic hysteresis and the daily thermal cycles, potentially affecting the behavior of the FBGs sensorized mat.

Also the duration of the threshold exceedance can be efficiently used in order to reduce the possibility to have false alarms due to random noise. Nevertheless, in general, to reduce the possibility of false alarms, a more sophisticated signal processing strategy should be adopted to infer more detailed information on the intruder characteristics. Furthermore, dedicated studies on targets classifications are needed to improve the recognition capabilities (even if this is out the scopes of this framework mainly focused on the technological development and demonstration of the sensing system).

**Figure 12.**
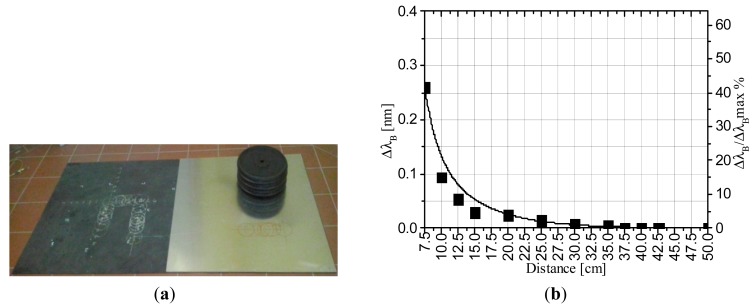
(**a**) Picture of the mat with aluminum plate; (**b**) Bragg wavelength shift *versus* the distance from the FBG fitted with an exponential decay.

Finally, here we report on recent results obtained with the intent of optimizing the performance of the proposed sensing mat. Specifically, to decrease the number of FBGs per area unit, we investigated the possibility of increasing the detection zone of each FBG using an aluminum plate on the rubber mat. Thus, we simply superimposed an aluminum plate with an area of 1 × 1 m^2^ and a thickness of 1.5 mm on the rubber mat (see Figure 12a) and reapplied the 60-kg static loads at difference distances from the FBG.

Figure 12b shows the Bragg wavelength shift *versus* the distance from the FBG under test conditions. For the aluminum-covered mat, the amplitude of the wavelength shift decreases rapidly with positioning of the load at different distances from FBG. The wavelength shift indeed exponentially decays for increasing distances. Nevertheless, in this case, the decay is slightly slower because the wavelength shifts are greater for increasing distances. This result can be attributed to the rigidity of the plate, which distributes the load on a wider surface, improving the sensitivity of the FBG to loads located far away. Again, if we refer to the threshold of 5 pm to establish the limit of operation of a single FBG, we identify a separation length of approximately 33 cm, which implies a number of FBGs for the area unit of approximately 2.3 per square meter (instead of four FBG/m^2^).

Although significant for large areas, the beneficial effect of the reduction in the number of FBGs for unit area should obviously be evaluated by taking into account the additional costs due to the use of a rigid plate and the relative installation costs.

### 4.2. Acceleration Measurements

**Figure 13.**
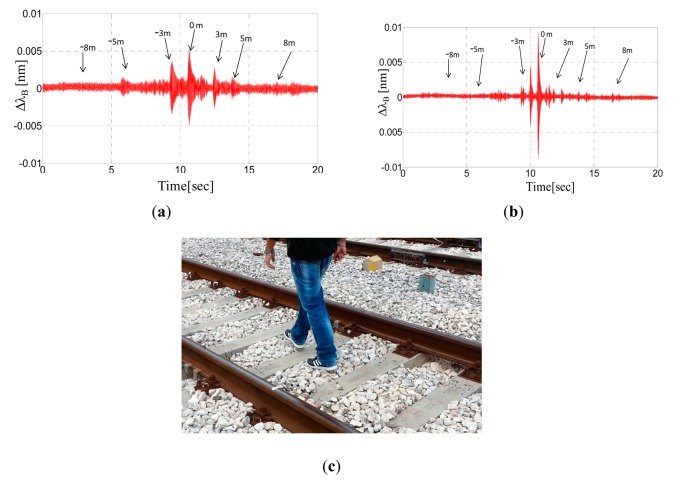
(**a**) Bragg wavelengths of the accelerometers *versus* time during the intruder's walk for the FBG labeled K1; (**b**) for the FBG labeled K2; and (**c**) the intruder's walk between the rails.

Figure 13a,b display the Bragg wavelengths shifts of the two accelerometers (labeled K1 and K2, respectively) *versus* time when the intruder walks between the rails of the track (see Figure 13c). The arrows indicate the correspondence between the time and the distance from the sensors as retrieved with the support of a camera that recorded the walk. It is possible to note that the accelerometer response is characterized by peaks due to the intruder's footsteps. These peaks are quite clearly distinguishable up at to 5 m of distance from both sides.

Overall, the amplitude of each peak is related to the distance of the intruder's step from the accelerometer. Nevertheless, differences in the response amplitudes of two identical accelerometers can be observed and are attributed to the structural non-uniformity of the ballast. The temporal separation between adjacent peaks is obviously related to the speed of the walking intruder (approximately 1 m/s) and the increasing amplitude of successive peaks is due to the intruder approach.

**Figure 14.**
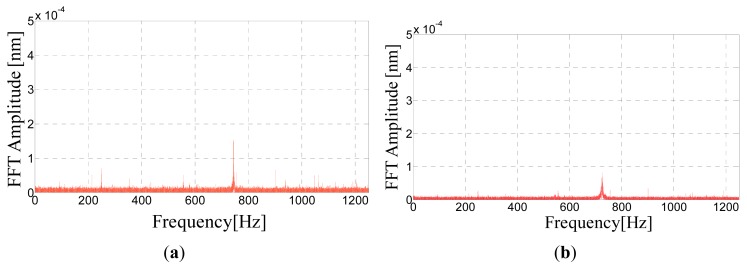
(**a**) FFT Amplitude of FBG accelerometers labeled K1; (**b**) FFT Amplitude of FBG accelerometers labeled K1.

Figure 14 shows the Fast Fourier Transform of the FBG accelerometer responses. The signals are primarily featured by one fundamental harmonic component at approximately 750 Hz corresponding to the resonance frequency of the accelerometer, which means that the accelerometer response is essentially due to excitation of its resonance frequency.

**Figure 15.**
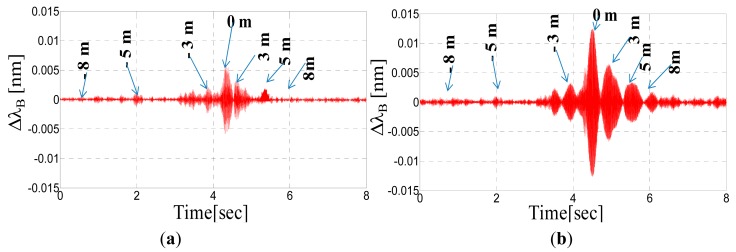
(**a**) Bragg wavelengths of the accelerometers *versus* time during the intruder's run between the rail-track for the FBG labeled K1; (**b**) for the FBG labeled K2.

We performed further intrusion tests through the rail track. Figure 15 shows the Bragg wavelength shift of the two accelerometers (labeled K1 and K2, respectively) *versus* time, when the intruder runs between the rails of the track. Peaks due to the footsteps can be clearly appreciated and no significant amplitude variations can be seen with respect to the walking intruder. The temporal separation of the peaks instead is reduced revealing a running speed of approximately 2.5–3 m/s.

**Figure 16.**
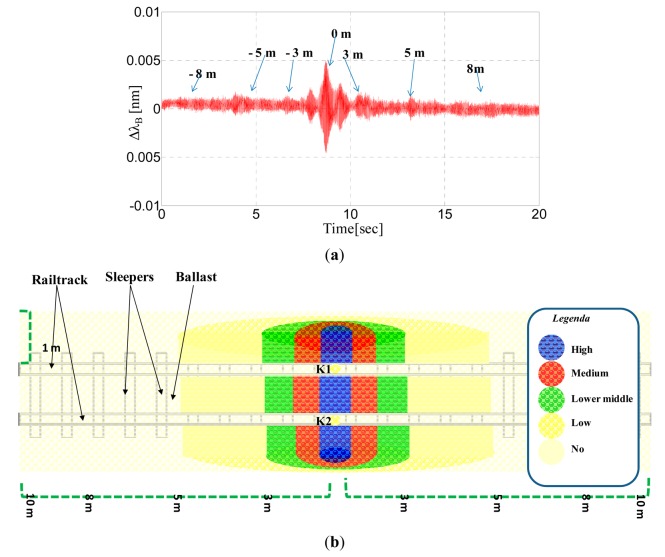
(**a**) Bragg wavelengths of the accelerometer K1 *vs*. time when the intruder walks outside and parallel at a distance of 80 cm from the rail (side A); (**b**) Schematics of the detection zone for FBG accelerometers sensing system.

Additionally, in Figure 16a, we show the response of the FBG accelerometers labeled K1 in the time domain when the intruder walks outside and parallel to the rail (side A) at a distance of 80 cm. In these experimental tests, human footsteps can be detected at up to three meters of distance, whereas for distances exceeding 80 cm, we found that the expected peaks are comparable with the noise floor.

Figure 16b illustrates the results of further and similar experimental tests using a colored representation. The schematics, although qualitative, illustrate the detection zone of the sensing system and its ability to reveal the presence of a man walking near the rail track access zone.

Finally, it is worth remarking that in this configuration, the proposed sensing system does not exploit “human signatures” to discriminate among different events [9], but the detection is generally associated with the intensity of the induced vibration. Nevertheless, the FBG accelerometers are able to detect a walking human and are thus suitable to reveal “intrusion” through the perimeter to be protected. Additionally, optimization margins still exist because the applied accelerometers and the package have been not optimized for this specific application.

## 5. Discussion and Conclusions

In this work, we demonstrated a technological solution based on FBG sensors suitable for protecting railway areas from unauthorized access. Using the same enabling technology, we designed and developed an optical fiber sensing system composed of a network of FBG strain sensors bonded on a ribbed mat combined with FBG accelerometers sensors installed directly on the rail tracks.

We carried out several experimental trials to characterize the elastic response of the sensorized mat and determine the performance in terms of the sensitivity curve, response times, and required number of sensors per area unit. The experimental results demonstrate that a network with four equally spaced FBGs per square meter enables the real-time detection of a human walking on the mat. Moreover, we demonstrated that optimization of the number of FBG per area unit is possible by covering the mat with a rigid plate that guarantees a wider distribution of the applied load.

Furthermore, we installed two FBG accelerometers in proper packages under the rail tracks at the Maddaloni-Marcianise freight station, where we performed several walking tests. The accelerometer sensors detected a walking human on the rail tracks, demonstrating their ability to detect unauthorized access through the rail tracks. An intruder was also detected at 5 m of distance from the installed sensors and approximately 0.8 m away from the railroad area.

During the performed tests, both subsystems demonstrated to be always able to detect a human walking through the protected perimeter, without exhibiting false negative. Additionally the experimental results indicated that the sensing system is able to provide further information on the intrusion such as the intruder speed and direction. The presence of multiple sensors, such as those installed on the mat, potentially enables also the localization of the intruder and the crossing pattern recognition.

We did not carry out dedicated studies on the sensing system's response to different moving targets. Nevertheless, in principle, we expect that also our sensing system could be subject to false positive as far as an interfering event induces an excitation on the sensors similar to the signals detected in presence of human walking.

In order to reduce the possibility of false alarms, high level signal processing strategies should be adopted also by exploiting and “fusing” data related to different spatial locations and time. Additionally (or alternatively), when the presence of false positive must be absolutely minimized, the proposed system could be used in combination with another intrusion detection system, such as a video camera system supported by automatic recognition algorithms. It is well known, indeed, that even if any intrusion detection system is subjected to its own false alarms, the combination of two systems with different technologies strongly improves the discrimination capability and reduces the false alarms [1].

In spite of the underlined potential limitations, the proposed FBG's integrated system offers a wide variety of features and benefits. Indeed, FBG sensors do not require electrical power, offer immunity to electromagnetic interferences, and high multiplexing capability allowing the deployment of a cost-effective interrogation strategy. Other basic components, constituting the integrated system, such as the rubber mat and the accelerometers packages, are low cost materials. Consequently, the startup, operation and maintenance costs of this integrated system are competitive or lower than other security methods.

Furthermore, the use of a technologically integrated solution paves the way for further functionalities integrated into the same platform for other possible security applications in the railway field. From the perspective of using a unique multifunctional technological platform, it is worth mentioning that FBG sensors have been widely used to perform smart diagnostics in such railway applications as switch monitoring, axle counting, weighing in motion, and wheel flat detection [12–15].
